# Promoting Research, Awareness, and Discussion on AI in Medicine Using #MedTwitterAI: A Longitudinal Twitter Hashtag Analysis

**DOI:** 10.3389/fpubh.2022.856571

**Published:** 2022-07-01

**Authors:** Faisal A. Nawaz, Austin A. Barr, Monali Y. Desai, Christos Tsagkaris, Romil Singh, Elisabeth Klager, Fabian Eibensteiner, Emil D. Parvanov, Mojca Hribersek, Maria Kletecka-Pulker, Harald Willschke, Atanas G. Atanasov

**Affiliations:** ^1^College of Medicine, Mohammed Bin Rashid University of Medicine and Health Sciences, Dubai, United Arab Emirates; ^2^McMaster University, Hamilton, ON, Canada; ^3^MYD Health, New York, NY, United States; ^4^Faculty of Medicine, University of Crete, Heraklion, Greece; ^5^Department of Internal Medicine, Allegheny General Hospital, Pittsburgh, PA, United States; ^6^Ludwig Boltzmann Institute for Digital Health and Patient Safety, Medical University of Vienna, Vienna, Austria; ^7^Division of Pediatric Nephrology and Gastroenterology, Department of Pediatrics and Adolescent Medicine, Comprehensive Center for Pediatrics, Medical University of Vienna, Vienna, Austria; ^8^Department of Translational Stem Cell Biology, Research Institute of the Medical University of Varna, Varna, Bulgaria; ^9^Institute for Ethics and Law in Medicine, University of Vienna, Vienna, Austria; ^10^Department of Anaesthesia, Intensive Care Medicine and Pain Medicine, Medical University of Vienna, Vienna, Austria; ^11^Institute of Genetics and Animal Biotechnology of the Polish Academy of Sciences, Warsaw, Poland

**Keywords:** social media, twitter, education, artificial intelligence, science communication

## Abstract

**Background:**

Artificial intelligence (AI) has the potential to reshape medical practice and the delivery of healthcare. Online discussions surrounding AI's utility in these domains are increasingly emerging, likely due to considerable interest from healthcare practitioners, medical technology developers, and other relevant stakeholders. However, many practitioners and medical students report limited understanding and familiarity with AI.

**Objective:**

To promote research, events, and resources at the intersection of AI and medicine for the online medical community, we created a Twitter-based campaign using the hashtag #MedTwitterAI.

**Methods:**

In the present study, we analyze the use of #MedTwitterAI by tracking tweets containing this hashtag posted from 26th March, 2019 to 26th March, 2021, using the Symplur Signals hashtag analytics tool. The full text of all #MedTwitterAI tweets was also extracted and subjected to a natural language processing analysis.

**Results:**

Over this time period, we identified 7,441 tweets containing #MedTwitterAI, posted by 1,519 unique Twitter users which generated 59,455,569 impressions. The most common identifiable locations for users including this hashtag in tweets were the United States (378/1,519), the United Kingdom (80/1,519), Canada (65/1,519), India (46/1,519), Spain (29/1,519), France (24/1,519), Italy (16/1,519), Australia (16/1,519), Germany (16/1,519), and Brazil (15/1,519). Tweets were frequently enhanced with links (80.2%), mentions of other accounts (93.9%), and photos (56.6%). The five most abundant single words were AI (artificial intelligence), patients, medicine, data, and learning. Sentiment analysis revealed an overall majority of positive single word sentiments (e.g., intelligence, improve) with 230 positive and 172 negative sentiments with a total of 658 and 342 mentions of all positive and negative sentiments, respectively. Most frequently mentioned negative sentiments were cancer, risk, and bias. Most common bigrams identified by Markov chain depiction were related to analytical methods (e.g., label-free detection) and medical conditions/biological processes (e.g., rare circulating tumor cells).

**Conclusion:**

These results demonstrate the generated considerable interest of using #MedTwitterAI for promoting relevant content and engaging a broad and geographically diverse audience. The use of hashtags in Twitter-based campaigns can be an effective tool to raise awareness of interdisciplinary fields and enable knowledge-sharing on a global scale.

## Introduction

Although in its early age, artificial intelligence (AI) is increasingly centered in online discussions surrounding medical practice and healthcare delivery. Several analyses indicate healthcare practitioners' and medical students' high interest in the utility of AI within these domains (1–10). Exponential increases in associated publications serve concomitantly as a reflection of and contributor to interest in the field. A PubMed search with the MeSH term “machine learning” (a progressively developing subset of AI) demonstrates this trend over the past decade, with just 49 records in the year 2010 and 8,408 publications in 2020. However, many healthcare practitioners and medical students report their lack of AI awareness (6–10)—this might be due to a limited understanding of benefits, limitations, societal implications, AI's current state, and future prospects. Furthermore, survey reports from Santos et al. (7) demonstrated that undergraduate medical students from several German medical universities were far more likely to have learned about AI from social media than university lectures, outlining the increasing educational significance of social media in this dynamically developing area.

The microblogging platform, Twitter, plays an integral role in the dissemination of news and literature. Healthcare practitioners are increasingly present on social networking sites, including Twitter, to interact with patients and peers, collaborate, and for the reciprocal exchange of ideas and information (11). Twitter consists of user-generated content containing up to 280 characters, called “tweets”, which can be publicly read and shared. Tweets may include images and links to research, events, and online resources. To aggregate content within a specific subject domain, users may include keywords preceded by a pound sign (#) as a “hashtag” in tweets. Several studies have demonstrated hashtags' utility in amplifying content associated with various topics and events. The inclusion of hashtags in tweets facilitated the promotion of disease-specific tweets (12–15), Twitter-based chats and journal clubs (16–19), and meeting or conference-related content (20–28). Hashtags have also been previously demonstrated as an effective tool for content-specific education (13, 29).

In this study, we aimed to evaluate the achieved outreach (defined as the act of reaching out to the Twitter-community, quantified by engagement metrics such as number of impressions and tweets, which were used as primary outcome measures) of a Twitter-based campaign using the hashtag #MedTwitterAI to promote research, events, and resources at the intersection of AI and medicine for the online medical community.

## Materials and Methods

### Campaign Development and Outreach

Registration of #MedTwitterAI as part of the Symplur healthcare hashtag project (29) was completed on April 19th, 2019 (30). Users were asked to include #MedTwitterAI in tweets sharing literature, resources, events, or discussion prompts associated with AI and medicine/healthcare. Promotion of the campaign involved engaging Twitter users with perceived interest in the subject (identified by their biographies and tweeting activity) by retweeting content, commenting on posts, Twitter-based chats, participation in live discussions, and a Twitter list. The Twitter list (31) was created on 17th October, 2020 and included 62 individual or organizational accounts which were determined by the authors to actively share relevant content associated with AI applications in medicine to aggregate associated tweets and further amplify the hashtag's visibility. To promote the visibility of relevant tweets, these users were engaged by the authors with direct messages, tweet mentions, tweet comments or quote retweets.

### Outcome Measures

To ensure the uniqueness of the hashtag #MedTwitterAI, Twitter search was performed before the start of the campaign. A project-related account dedicated to the project was not used, but instead selected relevant content was posted on Twitter by the campaign participants (the authors of the present manuscript). The project participants had liberty to personally select content to be posted by them, as well as to interact (by retweets or comments) with relevant content posted by other users, with the overall guiding principle being to focus on high-standard science-based content in the English language that is related to the applications of AI in medicine. Examples of shared content included dissemination of relevant conference information [Example: Stanford virtual conference with focus on COVID-19 and AI—(32)], scientific publications (Example: High-dimensional hepatopath data analysis by machine learning for predicting HBV-related fibrosis https://twitter.com/_atanas_/status/1367064744524460035), and educational opportunities [Example: The Imaging AI Certificate program of the Radiological Society of North America (33)].

### Data Extraction

The Symplur Signals research analytics tool was used to conduct an extensive assessment of tweets containing #MedTwitterAI in the timeframe ranging from 26th March, 2019 to 26th March, 2021. Symplur Signals represents a well-established hashtag analysis tool that that allows long-term tracking of tweets containing specific hashtags pre-registered with the Symplur healthcare hashtag project (29). The analysis performed with Symplur Signals assessed the cumulative number of tweets, impressions (i.e., views of tweets), and unique users sharing tweets containing #MedTwitterAI (including user categorization to specific healthcare stakeholder groups). All tweets containing the hashtag #MedTwitterAI were analyzed with Symplur Signals, without any restrictions on language, location of users or other parameters. Primary outcome measures for the achieved outreach (defined as the act of reaching out to the Twitter-community) and awareness (defined as bringing relevant information and knowledge to the Twitter-community) were the number of tweets and impressions. Additional metrics included in the analysis were the content incorporated in these tweets (e.g., links, mentions of other accounts, photos), co-occurring hashtags, tweet languages, and geolocation trends.

### Data Cleaning and Analysis

**Figure 1:**
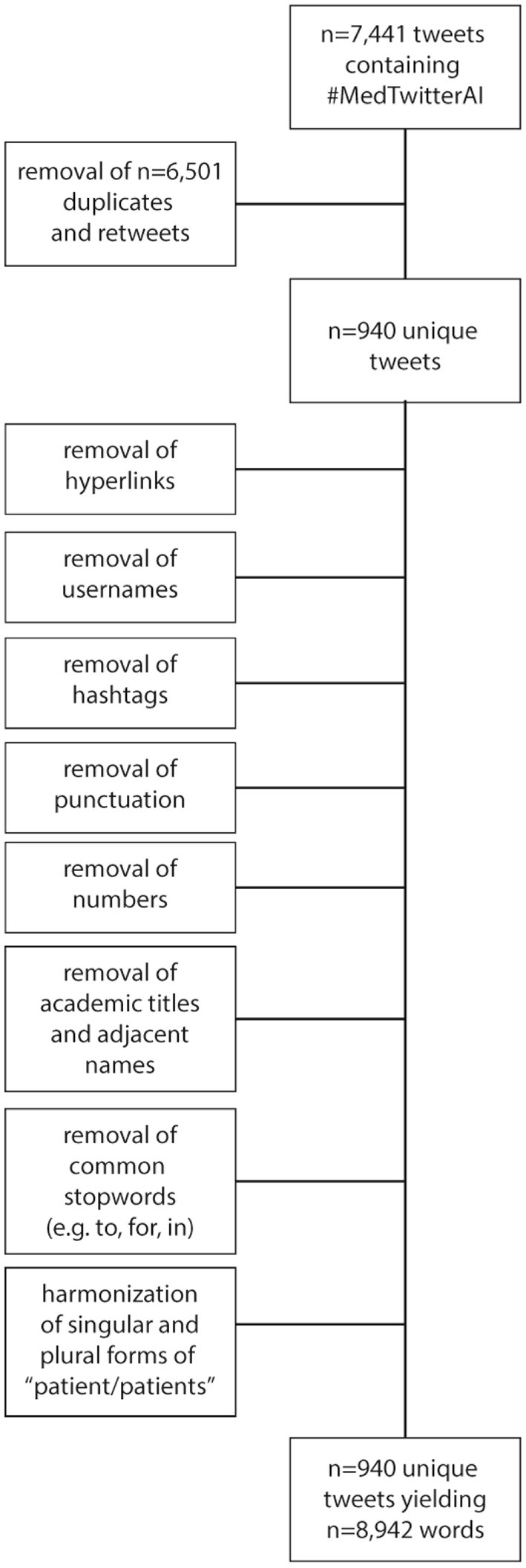
Flowchart depicting clean-up processing of tweets for sentiment analysis and Markov chain display.

For further identification of important emerging themes within the #MedTwitterAI identifier, we retrieved all respective tweets and submitted them to a natural language processing analysis utilizing R software [R Core Team 2020, (34)]. To gain accurate results for term frequency, sentiment analysis, and Markov chain display, the tweets were processed first. As displayed in Figure 1, this included removal of retweets, duplicate tweets, hyperlinks, usernames, hashtags, punctuation, numbers, common stop words (e.g., to, for, in), academic titles, and names. Furthermore, occurring singular and plural forms of the word “patient/patients” were harmonized to their plural forms. Other words were not harmonized to preserve meaning. Stop words were retrieved from the R package tidytext (35) which includes a large stop word database from three different English lexica. After clean-up processing, calculations of term frequency of single words, n-grams (bigrams, trigrams) as absolute frequencies, sentiment analysis, and Markov chain display of bigrams occurring more than 3 times (due to reasons of graphical display), were conducted. Sentiment analysis rates each word positive or negative in accordance with its declaration in a sentiment lexicon. For this analysis we utilized the “Opinion Mining, Sentiment Analysis, and Opinion Spam Detection” lexicon by Minqing and Liu (36) by automatically rating occurring words in accordance with their associated sentiment utilizing the R package tidytext (35). The Markov chain network graphs represent bigrams occurring more than 3 times, with arrows following the starting word of the most common bigrams to the most common words following it, colored by frequency of occurrence of the respective chain of words and were realized with the R package tidytext. In addition, all hashtags from the processed cleaned tweets were extracted and analyzed for frequency of occurrence. Visualization was accomplished as bar charts and Markov chain network graphs. As the sentiment analysis was not the primary focus of this research and of explorative character only in addition to a high quantity of occurring words and to these methods having been studied and published before (37), no human verification was conducted to determine the accuracy of this sentiment lexicon.

### Ethical Approval and Informed Consent

Ethical approval was not required for this study since this campaign was focused on information published in the public domain and the identity of individual users is not disclosed.

## Results

### Headings

**Figure 2:**
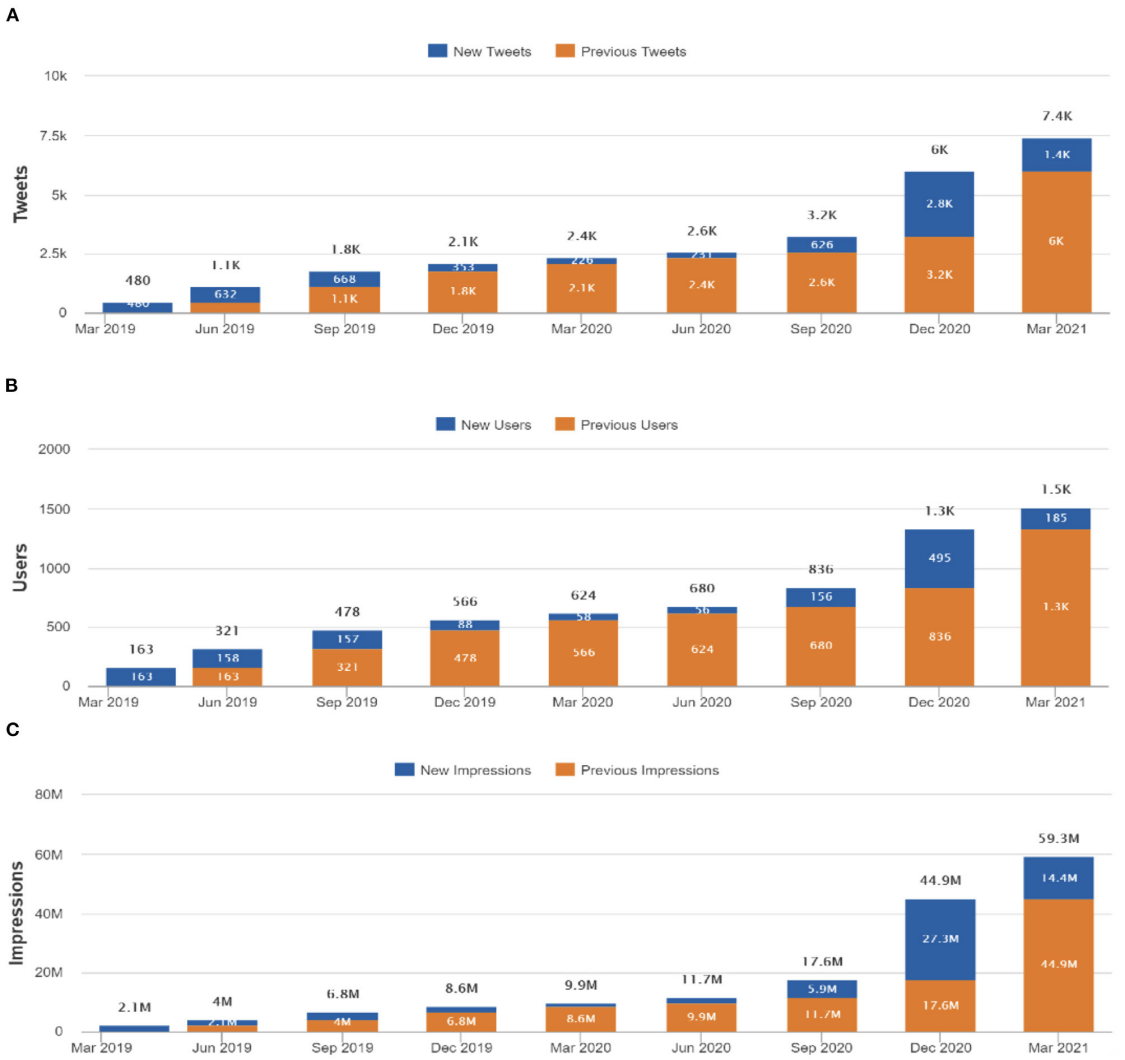
Cumulative increase in number of **(A)** #MedTwitterAI tweets, **(B)** unique users, and **(C)** impressions from 26th, March 2019 to 26th March, 2021 at 3-month time intervals. The indicated parameters were analyzed using the Symplur Signals hashtag analytics tool.

**Figure 3:**
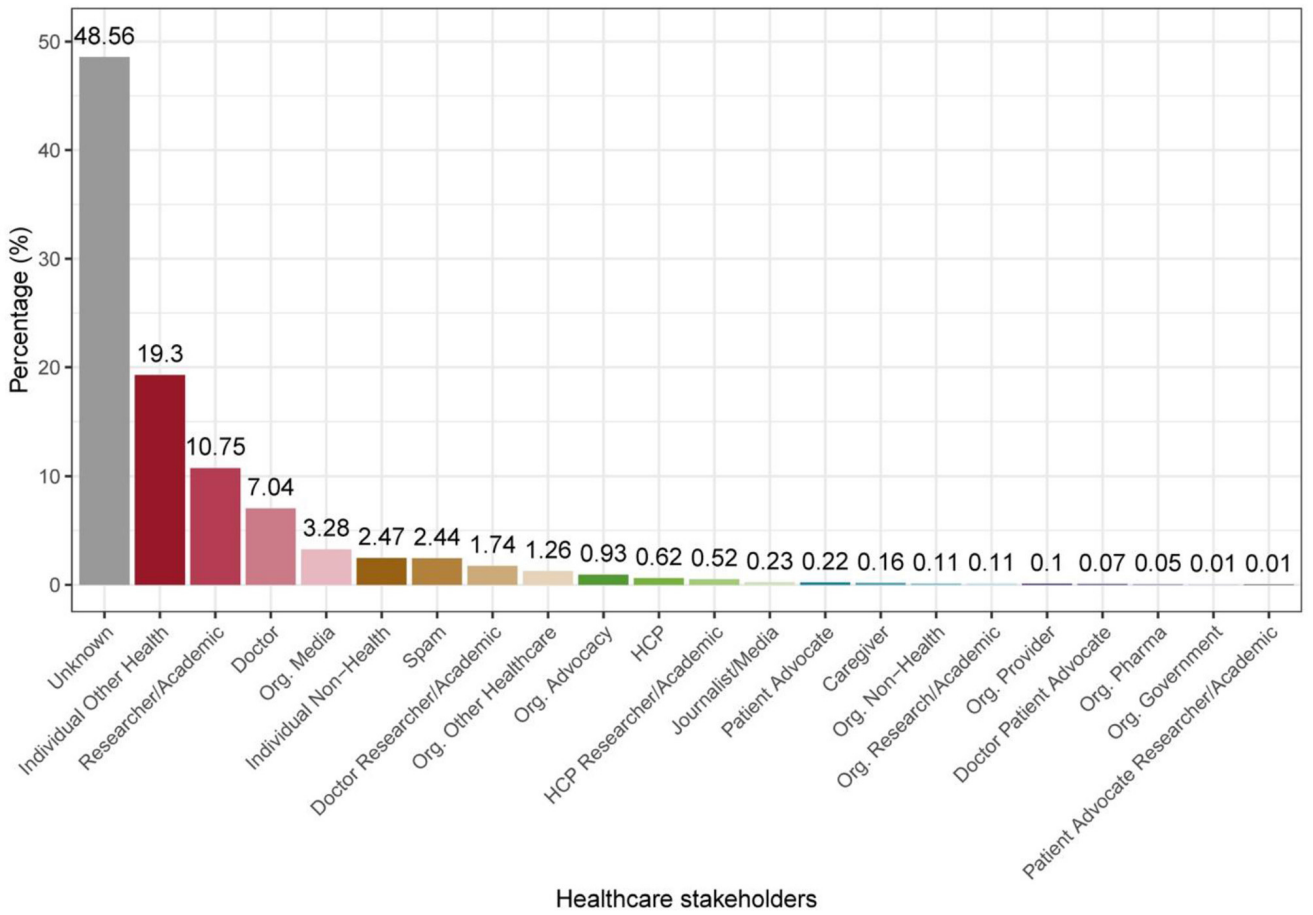
Percentage of #MedTwitterAI-posting healthcare stakeholders (data derived from Symplur Signals). Definitions according to the glossary by Symplur (listed alphabetically; note: as evident from the image approximately half of the accounts did not provide sufficient information to be categorized and are thus labeled as “Unknown”): Doctor: Those believed to be licensed, MDs, DOs, PhDs who bill directly for services. Also includes medical residents; HCP: Those believed to be other healthcare professionals (i.e., nurses, dietitians, respiratory therapists, nurses, pharmacists, etc.); Patient Advocate: Person who publicly self-identifies in their Twitter bio as a patient advocate for a specific disease or condition; Caregiver: A professional caregiver or a person who is currently or has been a caregiver of a family member or other closely associated individual; Researcher/Academic: Person who is working in the field of health-related research and/or academia. Note: A PhD who does not treat patients falls in this category; Journalist/Media: Person whose profession is journalism or other news-related media. Doctors who are editors of journals do not get this label; Individual Other Health: Person working in the healthcare industry in a non-clinical role; Individual Non-Health: Person not known to be directly working in the healthcare industry; Org. Provider: Inpatient facilities, medical groups, labs, imaging centers, and other outpatient facilities; Org. Research/Academic: Accredited schools of higher learning (i.e., universities, colleges, etc.) and healthcare research institutions/centers; Org. Government: Government accounts at local, state and national levels; Org. Advocacy: An organization focused on a specific set of health issues or medical specialty for the purpose of support, guidance, and education; Org. Pharma: All organizations in the pharmaceutical industry; Org. Media: All organizations whose primary purpose is publishing or broadcasting; Org. Other Healthcare: Organizations fulfilling roles within the healthcare industry but not providing direct clinical care; Org. Non-Health: All organizations not falling into an established healthcare category; Spam: Accounts reported to be associated with spam; Unknown: Not categorized.

The hashtag #MedTwitterAI was first included in a tweet for the Twitter-based campaign on 26th, March 2019 and activity was monitored until 26th March, 2021. Over this 2-year period, 7,441 tweets containing #MedTwitterAI were shared by 1,519 unique Twitter users which generated 59,455,569 impressions. Figure 2 plots cumulative number of: (A) tweets containing #MedTwitterAI, (B) unique users, and (C) generated impressions at 3-month intervals. In the first year of the campaign (from 26th March, 2019 to 26th March, 2020), 2.4 thousand tweets were recorded from 624 unique users, which generated 9.9 million impressions. An additional 5 thousand tweets, 895 unique users, and 49.4 million impressions were observed between 26th March, 2020 and 26th March, 2021. From the 1,519 total unique users, 69.6% generated one tweet, 12.7% two, and 17.7% three or more tweets containing the #MedTwitterAI hashtag. Concerning percentage-distribution of #MedTwitterAI-posting users in different healthcare stakeholders categories (data derived from Symplur Signals, with the classification being based on information provided in the Twitter biographies of the users), the three biggest groups of contributors were Individual Other Health (19.3% of all users; 37.5% of total classifiable users), Researcher/Academic (10.75%; 20.9% of total classifiable users), and Doctor (7.04%; 13.7% of total classifiable users), with the full distribution in the 22 identified categories depicted in Figure 3 (note: 48.56% of the accounts did not provide sufficient information to be categorized and are thus labeled as “Unknown”). Table 1 depicts the top 10 locations of users that tweeted content containing #MedTwitterAI who provided location data in their profiles. The most common locations of users were the United States (378), United Kingdom (80), Canada (65), India (46), Spain (29), France (24), Italy (16), Australia (16), Germany (16), and Brazil (15). Tweets with a detected language were mainly posted in English (7,055) followed by Spanish (11), French (6), Turkish (6), and Portuguese (3).

**Table 1:** Top ten locations of users that included #MedTwitterAI in tweets from 26th, March 2019 to 26th March, 2021.

**Country**	**Users**
United States of America	378
United Kingdom	80
Canada	65
India	46
Spain	29
France	24
Italy	16
Australia	16
Germany	16
Brazil	15

*Geolocation trends were tracked for users with location information provided in their Twitter profiles and analyzed using the Symplur Signals hashtag analytics tool*.

The #MedTwitterAI associated list amassed 162 followers by 26th March, 2021 from creation on 17th October, 2020 (31). Evaluation of viewership and amplification directly associated with this list could not be conducted for lack of accessible data.

**Figure 4:**
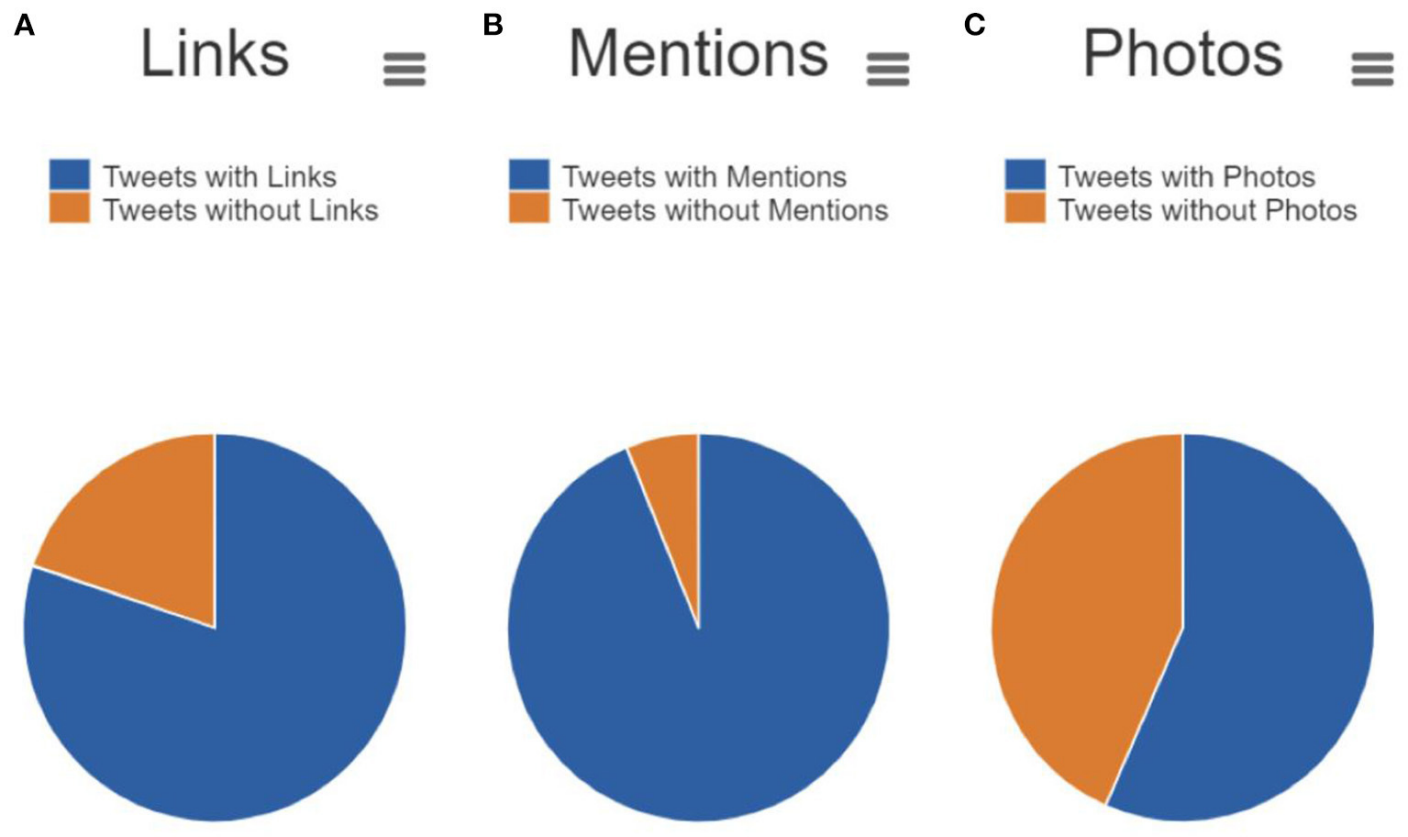
Proportion of #MedTwitterAI tweets containing **(A)** links (80.2%), **(B)** mentions (i.e., of other Twitter accounts) (93.9%), and **(C)** photos (56.6%) from 26th, March 2019 to 26th March, 2021. The indicated parameters were analyzed using the Symplur Signals hashtag analytics tool.

A content analysis of cataloged tweets is depicted in Figure 4. From the 7,441 recorded tweets, 80.2% contained links, 93.9% contained mentions of other accounts, and 56.6% contained photos. Common co-occurring hashtags included #AI (53.2%), #ML (27.6%), #DHPSP (26.2%), #ArtificialIntelligence (25.0%), #100DaysOfCode (21.9%), #MachineLearning (21.6%), #MedTwitter (13.8%), #DeepLearning (12.0%), #DigitalHealth (11.9%), and #HealthTech (8.5%).

**Figure 5:**
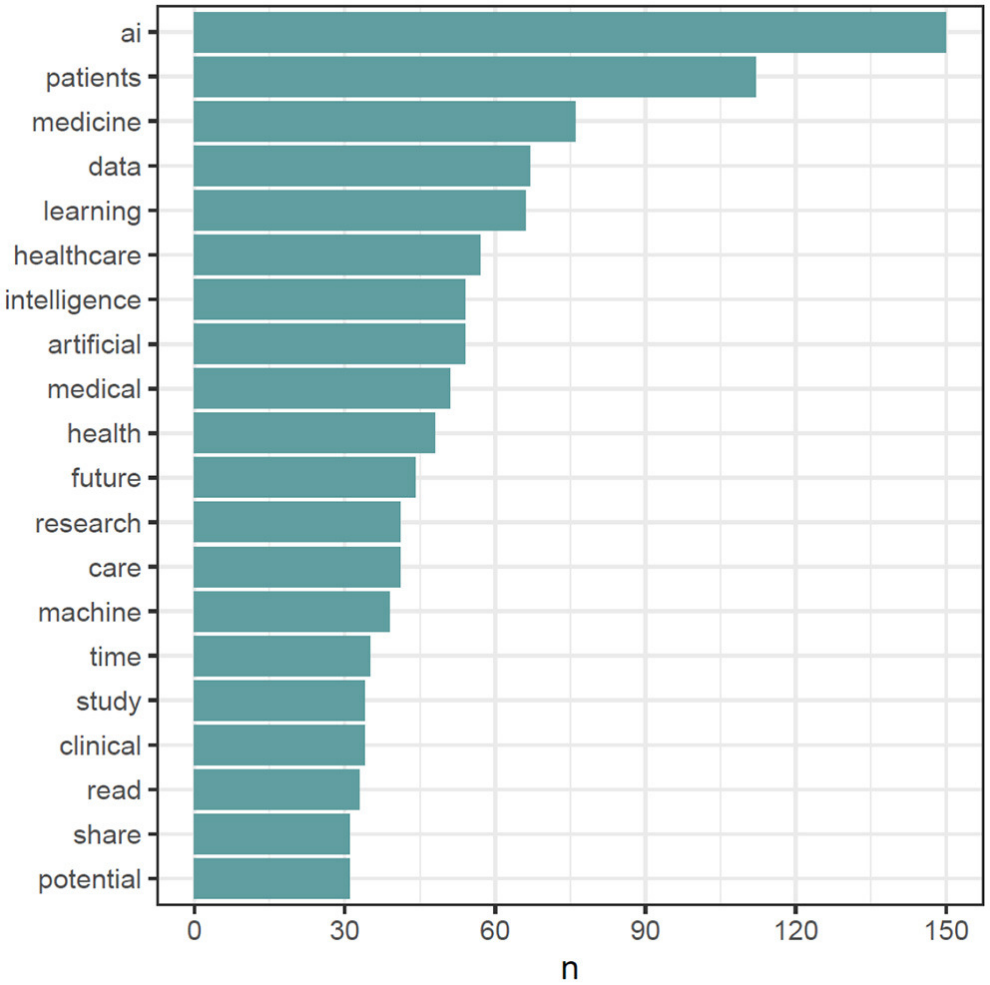
Top 20 most abundant single-words after preprocessing and cleanup, including removal of retweets, duplicate tweets, hyperlinks, usernames, hashtags, punctuation, numbers, common stop words (e.g., to, for, in), academic titles and name, and after harmonization of plural and singular forms of “patient/patients”.

**Figure 6:**
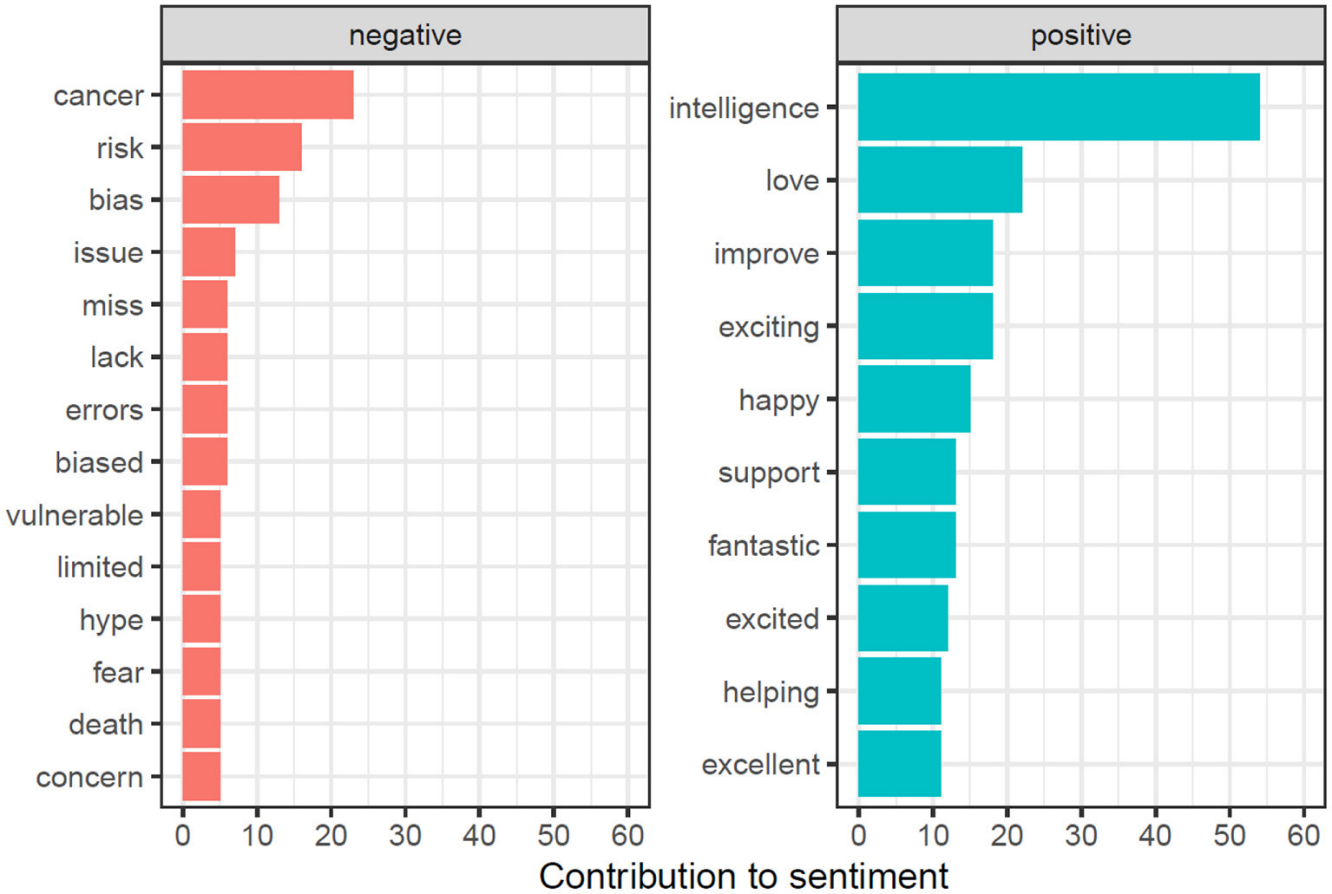
Sentiment analysis (at least five times mentioned) after preprocessing and cleanup, utilizing the “Opinion Mining, Sentiment Analysis, and Opinion Spam Detection” lexicon by Bing Liu and colleagues.

**Figure 7:**
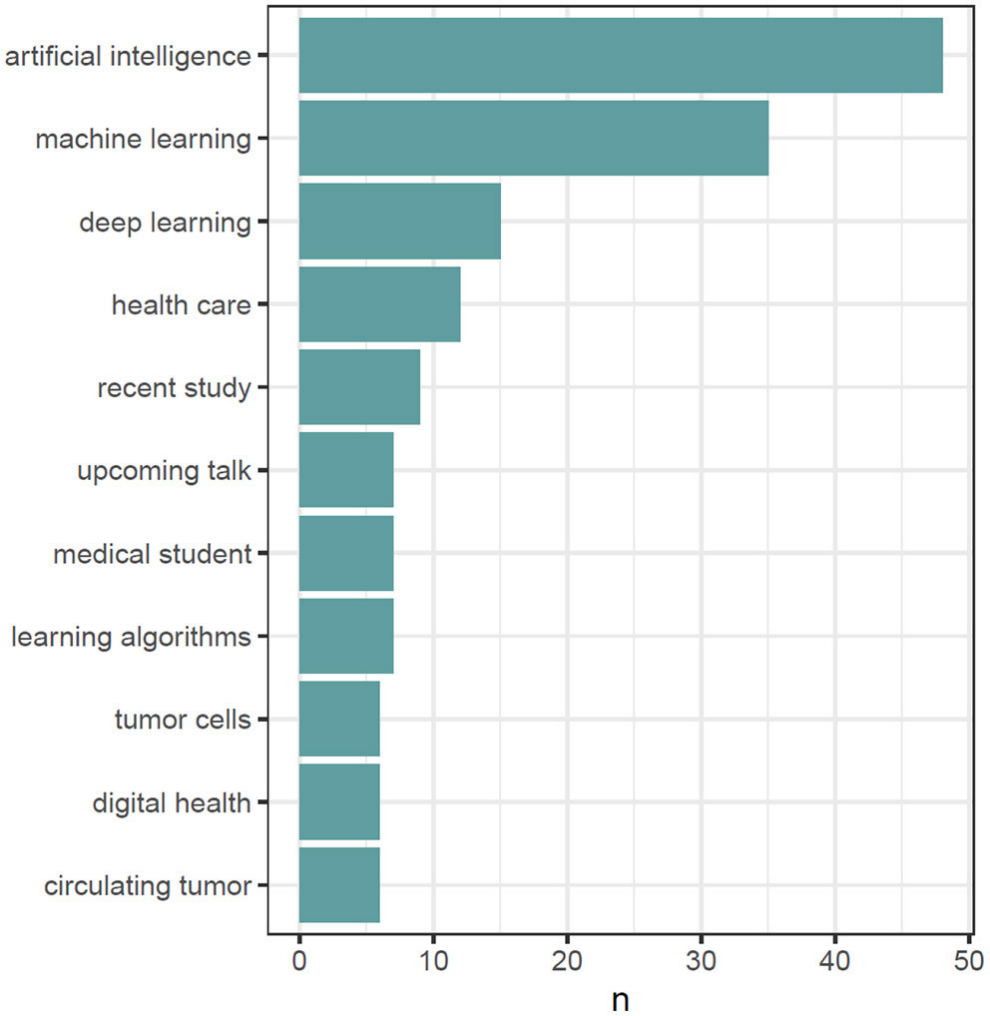
Top 10 bigrams (TOP x if ties) after preprocessing and cleanup, including removal of retweets, duplicate tweets, hyperlinks, usernames, hashtags, punctuation, numbers, common stop words (e.g., to, for, in), academic titles and name, and after harmonization of plural and singular forms of “patient/patients”.

**Figure 8:**
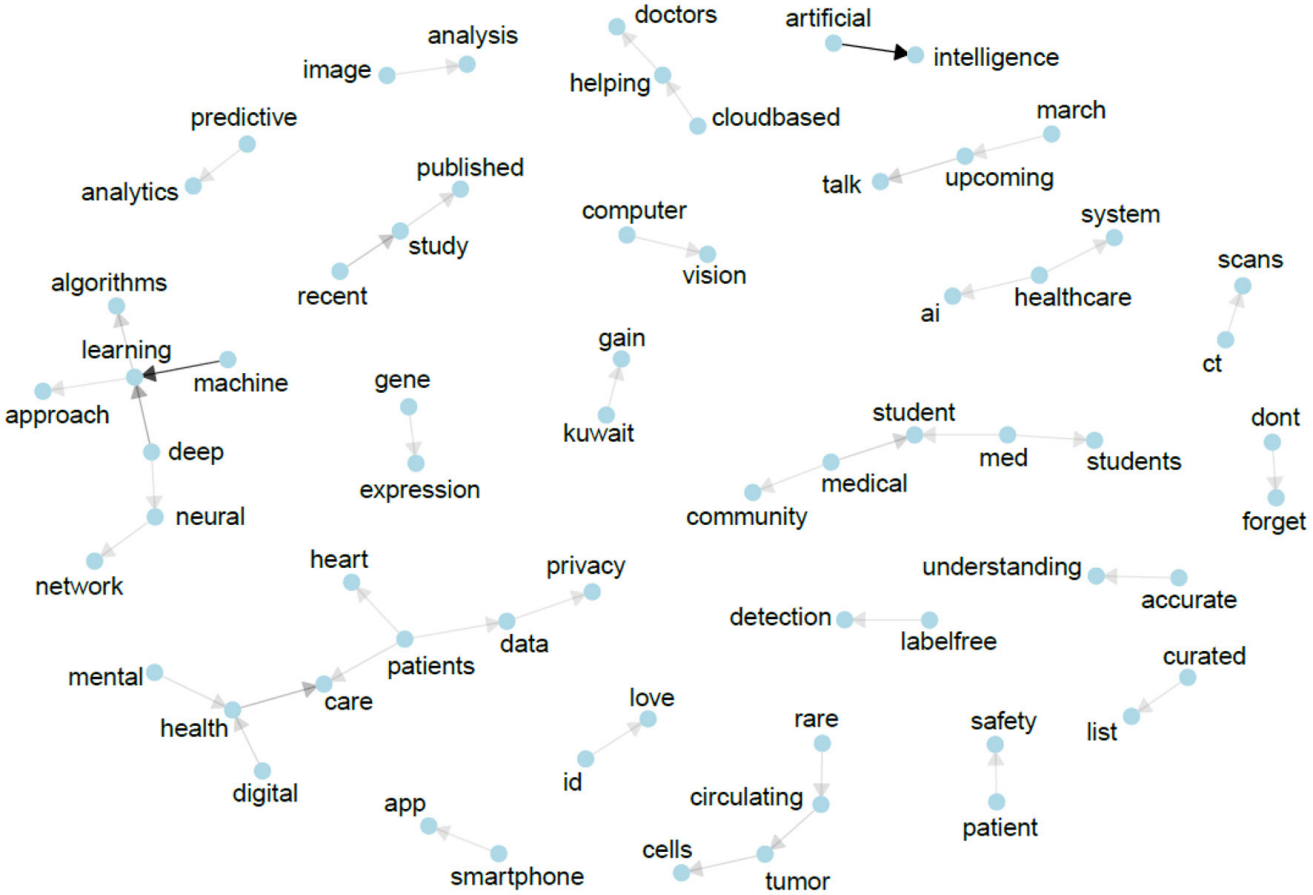
Markov chain of bigrams occurring > 3 times after preprocessing and cleanup. Arrows follow from the starting word of the most common bigrams to the most common words following it. Arrow color, from light gray to black, marks the frequency of occurrence.

**Figure 9:**
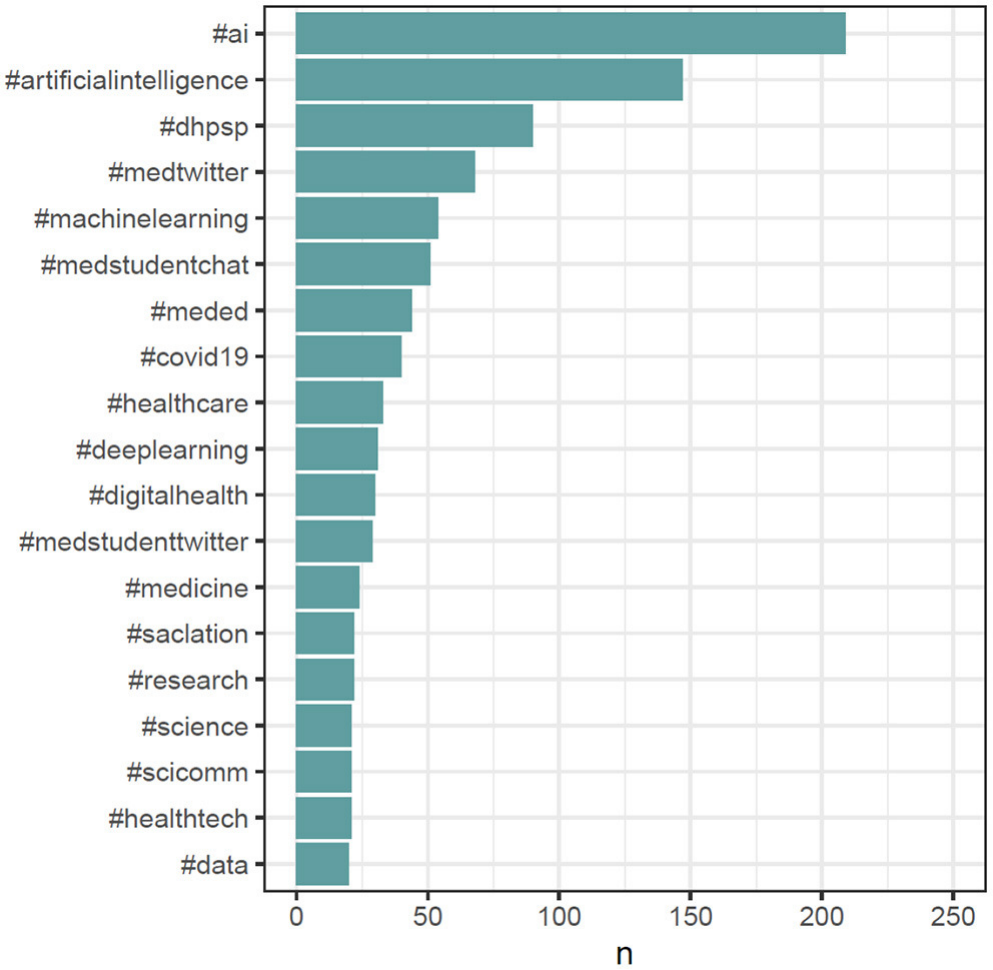
Top 20 hashtags included in tweets other than #MedTwitterAI.

For gaining deeper insights, the text of all #MedTwitterAI tweets was retrieved with Symplur Signals and submitted to a natural language processing analysis. After cleanup of the retrieved tweets, as described in Section Materials and Methods, a total of 3,826 unique words were identified from a total pool of 8,942 words (excluding stop words, double mentions of singular/plural etc.). A total of 417 unique hashtags from an initial pool of 2,050 hashtag mentions (without retweets) were identified. The most abundant single-word was the abbreviation “AI” referring to artificial intelligence, with a total of 150 mentions within the tweet's texts (Figure 5). Artificial and intelligence in their written-out form each appeared 54 times. Further highly abundant single-words were patients, medicine, data, and learning, with 112, 76, 67, and 66 mentions, respectively, as displayed in Figure 5. Figure 6 depicts frequency distributions of positive and negative sentiments within the analyzed tweet's texts, with an overall majority of positive single-word sentiments (e.g., intelligence, love, improve, exciting) with 230 positive and 172 negative sentiments with a total of 658 and 342 mentions of all positive and negative sentiments, respectively. The three most abundant negative single-word sentiments were cancer, risk and bias with 23, 16, and 13 mentions, respectively (details on the applied sentiment analysis approach are presented in Section Materials and Methods). Bigram analysis, as displayed in Figure 7 reveals the ten most mentioned bigrams to be artificial intelligence, machine learning, deep learning, and health care with a total of 48, 35, 15, and 12 mentions, respectively. The Markov chain depiction of bigrams occurring more than three times (Figure 8) unveils the context of these most common bigrams in several major groups, such as analytical methods (e.g., label-free detection, artificial intelligence, predictive analytics, image analysis, deep learning algorithms, machine learning algorithms/approach, deep neural networks) and medical conditions/biological processes (e.g., rare circulating tumor cells, mental health care, gene expression). Figure 9 displays the results of the tweet's hashtag analysis, with the most utilized hashtags being #ai, #artificialintelligence, #dhpsp, #medtwitter, and #machinelearning with 209, 147, 90, 68, and 54 mentions, respectively.

## Discussion

### Summary, Cross-Evaluation With Contemporary Literature

We performed a 2-year longitudinal analysis of the activity, users, and content associated with #MedTwitterAI using the Symplur Signals hashtag analytics tool and a natural language processing analysis. Starting at low basal levels, gradually increasing #MedTwitterAI use was ultimately reflected in 7,441 cumulative tweets, which generated 59,455,569 impressions. Content was produced by a geographically diverse group of 1,519 unique users. Most users generated one tweet during this period and content was mainly published in English. Tweets were frequently enhanced with links, mentions of other accounts, and photos. The high proportion of aggregated content containing external resources and mentions of other accounts demonstrates the knowledge-sharing conducted through this campaign. Furthermore, co-occurring hashtags indicated the engagement of various online communities (e.g., #MedTwitter, #DHPSP, #100DaysOfCode) and fields of interest (e.g., #MachineLearning, #DeepLearning, #DigitalHealth, #HealthTech).

Thorough analysis of the tweets including #MedTwitterAI using a natural language processing analysis revealed interesting patterns and emerging themes, both in the areas of medicine and technical topics (AI), showing that the abbreviation approach of the used hashtag (“Med” for medicine and “AI” for artificial intelligence) has been understood and taken up by the twitter community. Regarding different methods and medical conditions/biologic processes associated with artificial intelligence within the medical community, the analysis of rare circulating tumor cells, gene expression or mental health care, were among others, pivotal topics within the formed community. A qualitative review of the year before the conductance of this study assigned advancements of artificial intelligence to three major groups: artificial intelligence in medical diagnosis, psychiatry, and treatment (38). Similar to our study, prevalent topics of artificial intelligence application were related to cancer and image analysis (e.g., neural networks for the diagnosis of skin cancer on clinical images) or radiological imaging (e.g., deep learning algorithms of brain magnetic resonance imaging studies) to predict the diagnosis of autism in individual high-risk children (38, 39). Mental health in the context of digital patient care was a majorly discussed topic amongst tweets containing #MedTwitterAI. This was also evident in the qualitative review by Erwin Loh, where the prediction of suicide by machine-learning algorithms, machine learning models utilizing functional magnetic resonance imaging for the identification of patients with more severe negative and positive symptoms of schizophrenia or prediction of lithium response in bipolar patients were highlighted (38). However, in contrast to this review, our study did not yield the application of artificial intelligence in surgery, such as robotic surgical devices controlled by artificial intelligence for stitch-up of pig's small intestines or human teeth implantation (38).

Some tweets were also dedicated to sharing knowledge of recently published studies, upcoming talks or reaching out to medical students. As highlighted by others (37–39) and also discussed within the tweets of the #MedTwitterAI hashtag, education of medical students on this emerging topic should not be underestimated, and appropriate knowledge should be provided to future doctors that could possibly apply technology incorporating artificial intelligence directly to patients clinically.

Other Twitter participants actively engaged in technical talks on different analytical methods (e.g., machine learning algorithms, deep learning, neural networks, image analysis predictive analytics). However, as sentiment analysis demonstrated, overall engagement was mostly positive toward artificial intelligence within the medical field, focusing on improvement of these methodologies. This is in line with the attitudes of patients, physicians, and medical students in different medical fields (e.g., dermatology, neurosurgery) assessed in small survey studies, where attitudes were generally optimistic for improvement of clinical routine by the aid of artificial intelligence in certain tasks (7, 10, 40, 41). Artificial intelligence in medicine was also discussed critically within the discussion formed around #MedTwitterAI, especially regarding risk of bias and errors but also addressing patients' data privacy. Current reviews regarding ethics of artificial intelligence in medicine similarly identified the following ethical concerns of artificial intelligence application in medicine in the current literature: privacy, trust, accountability, and bias (42). However, deeper discussion of these topics is out of scope of this work, and the interested reader is referred to the respective literature, as for example discussed by Murphy et al. in BMC Medical Ethics 2021 (36). Since our study period also encompasses part of the COVID-19 pandemic, #COVID19 also appears within the 20 most abundant co-occurring hashtags. COVID-19 has emerged as an important topic to artificial intelligence in medicine as a recent review highlights, with several different applications amidst the COVID-19 pandemic (e.g., diagnosis, clinical decision making, therapeutics, public health applications) (43).

This Twitter campaign was unique given the interdisciplinary nature of subject, on the interface of computer science, engineering, and medical communities. Previous literature surrounding the use of hashtags in healthcare-associated content amplification tended to pertain to a single community (12–28). Several methodological distinctions are also apparent within our analysis. First, the timeframe of analysis (i.e., 2 years) was longer than studies of comparable subject, which provided data associated with hashtag growth across longer durations. Second, the breadth of analytical methods employed (e.g., geo-location trends, tweeting patterns, healthcare stakeholder categories, natural language processing analysis) provided unique insights toward a greater understanding of the campaigns. Third, as an original approach a Twitter list was created in attempt to further amplify and curate associated content.

Survey-based studies indicate the lack of AI familiarity amongst medical students and healthcare practitioners (6–10). Despite this, high expressed interest in AI amongst the medical community has been noted in several analyses (1–10). In addition, Santos et al. (7) report that undergraduate medical students from several German medical universities were far more likely to have learned about AI from social media than university lectures. The #MedTwitterAI Twitter-based campaign provided curated content to address the gap between interest and unfamiliarity. Given the campaign's substantive impact, our data clearly demonstrate that it efficiently engaged an array of diverse healthcare stakeholders, including medical doctors, patient advocates, caregivers, academic researchers, and journalists, among others (Figure 3). This broader stakeholder engagement is of great importance since it increases the potential that the dissemination of relevant AI-associated content on social media can contribute to knowledge translation, awareness, and may address hesitancy associated with AI implementations in practice. This campaign also contributes to the growing literature of hashtags' utility in amplifying content, which is of relevance to a general audience (anyone can create a hashtag for outreach of any purpose), as well as its applicability to interdisciplinary topics.

Till date, there has been no Twitter-based campaign in this domain that has reflected on the same scale of user interactions, yet the results are consistent with existing literature surrounding healthcare-centered hashtags. Salem et al. (15) similarly engaged a diverse group of stakeholders and promoted disease-related content using the hashtag #KidneyStones. Evaluation of #KidneyStones activity also demonstrated high proportions of tweets containing links and mentions. A high share of popular posts from a cardio-oncology Twitter Chat contained links to academic publications presenting relevant research results (16). As an example of Twitter hashtag analysis in the context of medical education, Rashid et al. (31) aggregated free open-access medical educational (FOAMed) resources for the online medical community using #FOAMed. Findings indicated that hashtags are successful in promoting curated educational content for healthcare-associated audiences (32). These findings provide a substantive basis for #MedTwitterAI's utility in increasing AI awareness amongst healthcare practitioners and medical students through social media.

### Stress and Limitations of the Study

One of the major strengths of this campaign is its systematic approach to knowledge-sharing. Individuals of varying geographic and academic backgrounds collaborated to promote research, events, and resources at the intersection of AI and medicine for the online medical community. Use of #MedTwitterAI engaged a diverse group of users and promoted awareness of an interdisciplinary and burgeoning field. However, we acknowledge certain practical limitations associated with this analysis' methodology. First, while all tweets which contained #MedTwitterAI posted during the 2-year timeframe were tracked, additional tweets, comments, and discussions which may have been facilitated by this campaign but did not directly contain #MedTwitterAI were not included. Accordingly, our analysis may not have captured the totality of #MedTwitterAI's impact. Second, Twitter also imposes several content restrictions for tweets (e.g., 280-characters, 4 photos) which may limit the ability to convey information effectively. Some users may be deterred from using the #MedTwitterAI hashtag as it contributes to a tweet's character count. Third, we used engagement metrics such as number of impressions and tweets as quantitative outcome measures of the outreach achieved by the Twitter campaign, however, these metrics do not provide information on how many of the users carefully read the provided information and acquired new knowledge.

Although liking a tweet can indeed lack consideration or be based either on personal friendship or on twitter content promotion strategies, retweeting and commenting reflect a level of understanding. The concept of the echo chamber may apply to retweeting without consideration (44). Nevertheless, during the last months Twitter has introduced a new function to address this issue. An automatic message asks the users to confirm whether they are aware of the content they are retweeting. Although it is not possible to measure and evaluate the intentions of every user, the platform's interface increases the possibility of conscious online interaction. Fourth, natural language processing analysis, especially n-grams, and sentiment analysis is generally limited by the absence of contextual valence. Typing errors were not corrected within this analysis, and synonyms were not harmonized, therefore some topics may be over- or under-represented within this analysis. And finally, as we did not primarily focus on sentiment analysis, which was of explorative character only, no human verification was conducted to determine the accuracy of the utilized sentiment lexicon. However, this sentiment lexicon and analysis method has been successfully utilized before (37).

### Future Research

Herein we provide several recommendations for future hashtag-based campaigns and associated analyses. The largest quarterly increase in recorded tweets, users, and impressions were observed for #MedTwitterAI between September 2020 and December 2020. During this period the #MedTwitterAI Twitter list was created (17th October, 2020), which may have correlated. Although direct assessments of the list's impact could not be readily made, future studies may evaluate the effectiveness of Twitter lists in promoting healthcare-associated content. In addition, online events based exclusively on Twitter (e.g., chats, journal clubs) or facilitated through different platforms (e.g., webinars, hackathons) may be held to enhance #MedTwitterAI outreach.

To further evaluate the effectiveness of hashtag-based knowledge-sharing, direct assessments (e.g., surveys, tests) of self-perceived familiarity or objective knowledge may be conducted on users engaging with content. Attai et al. (45) used surveys to demonstrate that engagement with the Breast Cancer Social Media Twitter support community (#BCSM) increased breast cancer patients' perceived knowledge; similar methodology may be employed to assess healthcare practitioners' and medical students' reported AI awareness after engagement with curated content. Assessments may also be conducted to determine if engagement with AI-based hashtags can influence attitudes toward AI adoption.

## Conclusion

An emerging body of evidence has reported the use of social media to share knowledge about common (COVID-19, kidney stones, incontinence), sensitive (infertility, cancer), and lesser-known health issues (cardio-oncology). The use of hashtags in Twitter-based campaigns can be an effective tool to raise awareness of interdisciplinary fields and enable knowledge-sharing on a global scale. The present study has focused on increasing awareness about AI, an emerging technology with a growing role in healthcare. So far, it has generated a noteworthy and diverse level of engagement on social media. It is of interest to observe whether the hypothesized awareness will last in time with polls and questionnaires and whether this will lead to a higher level of involvement of the users with AI in real life. Future studies can aim to amplify knowledge about other fields of health—technology such as digital health and bioprinting to increase healthcare workers' and patients' trust in them. The latter could be assessed in future studies by means of cross-sectional studies (polls, questionnaires) distributed via social media. Health bodies and physicians can use such cumulative insights to improve their social media and public outreach strategies. Finally, it is important to measure the generated awareness among students and trainees and monitor whether this will result in more individuals with a background in medicine pursuing further studies or research in engineering and informatics. Apart from social media polls, this can be thoroughly assessed in collaboration with universities and alumni associations.

## Data Availability Statement

The original contributions presented in the study are included in the article/supplementary material, further inquiries can be directed to the corresponding author.

## Author Contributions

FN, AB, RS, and CT prepared the first draft of this manuscript. AA and FE analyzed results. FN, AB, MD, RS, CT, EK, FE, EP, MH, MK-P, HW, and AA reviewed, edited, and approved the final manuscript. All authors contributed to the article and approved the submitted version.

## Conflict of Interest

The authors declare that the research was conducted in the absence of any commercial or financial relationships that could be construed as a potential conflict of interest.

## Publisher's Note

All claims expressed in this article are solely those of the authors and do not necessarily represent those of their affiliated organizations, or those of the publisher, the editors and the reviewers. Any product that may be evaluated in this article, or claim that may be made by its manufacturer, is not guaranteed or endorsed by the publisher.
